# A novel myeloid cell in murine spleen defined through gene profiling

**DOI:** 10.1111/jcmm.14382

**Published:** 2019-06-18

**Authors:** Ying‐Ying Hey, Terence J. O’Neill, Helen C. O’Neill

**Affiliations:** ^1^ Clem Jones Centre for Regenerative Medicine Bond University Gold Coast QLD Australia; ^2^ Big Data Centre Bond University Gold Coast QLD Australia

**Keywords:** dendritic cells, monocytes, myelopoiesis, spleen

## Abstract

A novel myeloid antigen presenting cell can be generated through in vitro haematopoiesis in long‐term splenic stromal cocultures. The in vivo equivalent subset was recently identified as phenotypically and functionally distinct from the spleen subsets of macrophages, conventional (c) dendritic cells (DC), resident monocytes, inflammatory monocytes and eosinophils. This novel subset which is myeloid on the basis of cell surface phenotype, but dendritic‐like on the basis of cell surface marker expression and antigen presenting function, has been tentatively labelled “L‐DC.” Transcriptome analysis has now been employed to determine the lineage relationship of this cell type with known splenic cDC and monocyte subsets. Principal components analysis showed separation of “L‐DC” and monocytes from cDC subsets in the second principal component. Hierarchical clustering then indicated a close lineage relationship between this novel subset, resident monocytes and inflammatory monocytes. Resident monocytes were the most closely aligned, with no genes specifically expressed by the novel subset. This subset, however, showed upregulation of genes reflecting both dendritic and myeloid lineages, with strong upregulation of several genes, particularly CD300e. While resident monocytes were found to be dependent on Toll‐like receptor signalling for development and were reduced in number in *Myd88*‐/‐ and *Trif*‐/‐ mutant mice, both the novel subset and inflammatory monocytes were unaffected. Here, we describe a novel myeloid cell type closely aligned with resident monocytes in terms of lineage but distinct in terms of development and functional capacity.

## 1 INTRODUCTION

Myelopoiesis is a regulated process of cell development leading to multiple cell types which contribute to both innate and adaptive immunity. A common myeloid progenitor in adult bone marrow gives rise to monocytes/macrophages, dendritic cells (DC) and granulocytes including neutrophils, eosinophils, basophils and mast cells.^1^, ^2^, ^3^ The spleen contains multiple myeloid subsets. While DC subsets are well characterized, the monocyte, macrophage and granulocyte lineages are less well defined.

Conventional (c) DC represent the main DC subset in murine spleen and have been further classified as functionally distinct CD8^+^ and CD8^−^ subsets.^4^ CD8^+^ cDC are distinct as CD11c^hi^CD11b^−^CD8α^+^MHCII^+^ cells, while CD8^−^ cDC have a CD11c^hi^CD11b^lo^CD8α^−^MHCII^+^ phenotype.^5^ These subsets differ in immune function, including cytokine production and ability to cross‐present antigen.^6^ The plasmacytoid (p)DC is another common splenic DC subset, existing as a plasmacytoid preDC in the steady‐state.^7^ Under inflammatory conditions, monocyte‐derived (mo)‐DC can develop when inflammatory stimuli recruit circulating classical or inflammatory monocytes from blood into spleen where they differentiate.^8^, ^9^, ^10^ Dendritic cells are located mainly within the white pulp region of spleen where immune responses against blood‐borne antigens and pathogens are initiated, while myeloid cells are primarily located within the red pulp region.

Spleen contains several subsets of tissue‐resident macrophages, namely marginal zone and marginal metaphyllic macrophages, red pulp macrophages and tingible body macrophages in the white pulp region.^11^ While the yolk sac origin of red pulp macrophages is understood,^12^, ^13^, ^14^ their relationship with splenic monocytes resident mainly in the red pulp region is still unclear. To date, no distinct phenotypic markers are available which can be used to distinguish red pulp macrophages from other myeloid subsets present in the red pulp. The distinction and lineage relationship between splenic monocytes and red pulp macrophages is not well understood.

Monocytes in blood and spleen are thought to derive from myeloid precursors in bone marrow.^7^ Two main subsets of circulating monocytes in blood are also present in spleen: the CX_3_CR1^lo^Ly6C^hi^ inflammatory or classical monocytes, and the CX_3_CR1^hi^Ly6C^−^ resident or non‐classical monocytes.^9^ Phenotypic identity of the two main monocyte subsets in spleen was recently clarified in this lab through marker phenotype and functional analysis.^15^, ^16^, ^17^ That study also classified splenic macrophages as CD11b^lo^ cells, with distinct macrophage subsets identifiable through staining with specific subset markers of SIGNR1, MOMA‐1, CD69 and F4/80.^17^

Under inflammatory conditions, both classical or inflammatory monocytes and non‐classical or resident monocytes are selectively mobilized from spleen to the site of inflammation. Here, classical monocytes clear damaged tissues, while non‐classical monocytes promote wound healing.^18^ Inflammatory monocytes can also home to sites of infection where they differentiate to give mo‐DC,^19^ while resident monocytes home to non‐inflammatory sites where they are thought to differentiate to give macrophages in some tissues, eg liver and spleen.^19^ Deployment of a reservoir of splenic monocytes was hypothesized as a mechanism for faster initiation of an immune response. Information on the function of non‐classical (resident) monocytes, and whether or not they differentiate to give macrophages within tissues, is still debatable. However, all tissue‐resident macrophages are not derived from resident monocytes and evidence for a yolk sac or foetal origin for tissue resident macrophages is forthcoming for some tissues.^14^

All evidence points to a major role for spleen in myelopoiesis. Our own previous work has identified a novel CD11b^hi^CD11c^lo^MHC‐II^−^ cell type in spleen. This was investigated as an in vivo equivalent to the named “L‐DC” subset of dendritic‐like antigen presenting cells produced in long‐term co‐cultures of haematopoietic progenitors over splenic stromal lines. The original studies on “L‐DC” produced in vitro described a dendritic‐like cell type which was distinct in terms of its phenotype as a CD11b^hi^CDllc^lo^MHC‐II^−^ cell antigen presenting cell, having very strong capacity to cross‐present antigen and to activate CD8^+^ cytotoxic T cells.^20^, ^21^, ^22^ It was predicted that such a cell type located in spleen may play a unique role in the induction of CD8 T cell immunity to blood‐borne antigens like pathogens and dead cancer cells (REF).^21^, ^22^ Their inability to activate CD4^+^ T cells would be consistent with their location and function at the level of the spleen and the blood stream because CD4+ T cell activation and cytokine production might be toxic.

In light of their unique functional capacity, studies were initiated to identify this specific cell type in spleen. The absence of specific markers made this process difficult. However, through a series of studies dissecting the myeloid cell populations in both murine and human spleen,^15^, ^23^ this novel splenic subset has been identified and analysed in terms of function equivalent to the in vitro‐derived cell type. The “L‐DC” subset in mice has been shown to be phenotypically distinct from the four splenic macrophage subsets,^17^ and both phenotypically and functionally distinct from the two splenic monocyte subsets.^15^, ^16^ It was also clearly distinguished from the main splenic DC subsets.^15^, ^16^, ^17^, ^24^ In terms of antigen presenting capacity, this novel subset is superior in capacity to cross‐present antigen to CD8 T cells and to activate cytotoxic function, although incapable of activating of CD4 T cells.^16^ These cells were shown by gene profiling to reflect a distinct cell type expressing genes common to both myeloid and dendritic lineages.^16^ This novel subset closely resembles dendritic‐like cells produced in vitro in long‐term cultures of spleen (LTC‐DC) and similar cells produced in co‐cultures of bone marrow progenitors over splenic stromal lines.^20^, ^25^, ^26^, ^27^ For this reason alone, it has been referred to as “L‐DC” in these studies.

Transcriptome analysis has been used here to analyse the relationship between this new subset and resident and inflammatory monocyte subsets in spleen, eosinophils and the CD8^+^ cDC and CD8^−^ cDC subsets. These subsets were isolated in line with a recently published subset identification method which redefined the resident monocyte subset in spleen, and also identified splenic eosinophils more fully.^15^ Gene profiling now clearly distinguishes this novel subset. While it is found to be closely related to resident and inflammatory monocytes, evidence presented here also distinguishes it as developmentally and functionally distinct.

## 2 MATERIALS AND METHODS

### 2.1 Animals

Specific pathogen‐free C57BL/6J, C57BL/6‐*MyD88* ^−/−^ (*MyD88^−/−^*) and C57BL/6‐*TRIF* ^−/−^ (*TRIF* ^−/−^) mice were obtained from the John Curtin School of Medical Research (JCSMR, Australian National University (ANU) and used at 4‐6 weeks of age. C57BL/6‐*MyD88* ^−/−^ *TRIF* ^−/−^ (*MyD88* ^−/−^ *TRIF* ^−/−^) mice were purchased from the Walter and Eliza Hall Institute (WEHI: Parkville, Victoria, Australia) and used at 4‐6 weeks of age. Animal experimentation was conducted according to protocols approved by the Animal Experimentation Ethics Committee at the ANU. Mice were sacrificed by cervical dislocation.

### 2.2 Cell preparation

Following dissociation of spleen tissue through crushing, T and B lymphocytes were depleted through column separation performed with MACS^®^ technology (Miltenyi Biotec: Auburn, California, USA). Cells were resuspended at 10^8^ cells/mL in MACS labelling buffer (2 mmol/L EDTA/0.5% BSA in PBS) and incubated on ice for 25 minutes with antibody: 0.2 µg biotinylated anti‐Thy1.2 antibody/10^8^ cells (T cells) and 0.25 µg biotinylated anti‐CD19 antibody/10^8^ cells (B cells) in 1 mL for 30 minutes at 4°C. Cells were washed by centrifugation, labelled by addition of 20 µL of anti‐biotin microbeads/10^8^ cells (Miltenyi) with incubation for 25 minutes on ice, followed by washing and resuspension in 500 µL of MACS labelling buffer. Separation involved use of LS columns (Miltenyi) in a SuperMACS II Separation Unit (Miltenyi), followed by washing thrice with 3 mL of MACS buffer. Unbound cells collected as flow‐through from the column were enriched for myeloid and dendritic cells.

### 2.3 Antibody staining and flow cytometry

Procedures for staining cells with antibodies for flow cytometry have been described in detail in earlier studies.^15^ Anti‐CD16/32 (FcBlock: 5 µg/mL) (Biolegend, San Diego, CA, USA) was used to block non‐specific antibody binding through Fc receptors. Fluorochrome‐ or biotin‐conjugated antibodies used were specific for CD11b, (M1/70), CD11c (N418), Ly6C (Al‐21), Ly6G (1A8), CD8 (53‐6.7), CD43 (1B11) and Siglec‐F (E50‐2440) (Biolegend). Propidium iodide staining (PI; 1 µg/mL) prior to flow cytometry was used to identify and gate live (PI^−^) cells. Flow cytometric analysis of labelled subsets was performed on a BD LSRII flow cytometer (Becton Dickinson, Franklin Lakes, NJ, USA). FACSDiva software (Becton Dickinson) was used to acquire data. FloJo software (Tree Star, Ashland, OR, USA) was used for data analysis.

### 2.4 Transcriptome analysis

Cell sorting of dendritic and myeloid subsets in spleen was performed as described previously^15^ with a FACSAria cell sorter (Becton Dickinson). RNA was extracted with an RNeasy mini kit (Qiagen, Clifton Hill, VIC, Australia) and used to prepare labelled cDNA for hybridization to genechips. Label preparation and hybridization was performed by Dr Kaiman Peng (Biomolecular Resource Facility, ANU, Canberra, ACT, Australia). The procedure followed the protocols for Applause WT‐Amp ST and WT‐Amp Plus ST RNA Amplification Systems posted on the website of NuGEN Technologies (San Carlos, CA, USA) (http://www.nugeninc.com/nugen/index.cfm/products/apl/applause-rna-amplification-systems/). Amplification of cDNA involved the SPIA amplification kit (NuGEN Technologies). The cDNA samples were fragmented and labelled according to the FL‐Ovation^TM^ cDNA Biotin Module V2 protocol (NuGEN Technologies), followed by hybridization to Mouse Gene 1.0 ST genechips (Affymetrix, Santa Clara, CA, USA). These were washed and stained with the Affymetrix fluidics station prior to scanning and analysis with an Affymetrix GeneArray^®^ Scanner. Duplicate arrays were prepared for each subset.

### 2.5 Microarray data analysis

Initial analysis was performed by Dr Stephen Ohms (Biomolecular Resource Facility, ANU). Scanned images of labelled genechips prepared in duplicate experiments were analysed with Partek to give average signal values and *P* values. Data files containing probeset numbers, gene descriptions, signal values and *P*‐values were prepared in text file format and subsequently exported into Microsoft Excel for principal components analysis. ANOVA was used for selection of genes up‐ or down‐regulated in pairwise comparison. Data mining was also used to assess the expression of genes linked to known functions in development or associated with distinct cell lineages. In addition, agglomerative hierarchical clustering (with the Lance–Williams dissimilarity formula)^28^ and heatmap analysis were performed with R project (http://www.r-project.org/). Results of hierarchical clustering analysis are presented as dendrograms on heatmaps.

### 2.6 Statistical analysis

Where indicated, data has been obtained from multiple animals, data are presented as mean ± SE for sample size (*n*). The Student’s *t* test has been used to determine significance (*P* ≤ 0.05).

## 3 RESULTS

### 3.1 Transcriptome analysis of splenic dendritic and myeloid subsets

The clearly defined populations of resident monocytes, inflammatory monocytes, eosinophils, CD8^+^ cDC and CD8^−^ cDC were sorted from spleens of C57BL/6J mice according to the staining procedure developed previously (Table 1).^15^, ^16^ This procedure initially gated out splenic macrophages on the basis of their CD11b^lo^CD11c^−^ phenotype as determined previously,^17^ and then identified monocytes and dendritic cells amongst the CD11b^hi^ population.^15^, ^17^ The phenotype of subsets under study here is summarized in Table 1. “L‐DC” are distinguished as a CD11b^hi^CD11c^lo^MHCII^−^Ly6C^−^Ly6G^−^ subset also shown to be CX3CR1^lo^CD43^lo^Siglec‐F^−^. They are distinct from eosinophils on the basis of CD11c^lo^ expression and lack of Ly6C and Siglec‐F expression. As CX_3_CR1^lo^Ly6C^−^CD115^−^ cells, they are distinct from CX_3_CR1^hi^Ly6C^lo^CD115^+^ resident monocytes. “L‐DC” are also distinct from CD11c^−^Ly6C^hi^ inflammatory monocytes. They are clearly distinct from cDC in that they lack MHCII expression and because they express CD43 and CX_3_CR1. High purity RNA was extracted from cells, converted to cDNA, biotin‐labelled and then hybridized to Murine Gene ST1.0 genechips (Affymetrix) for analysis of relative gene expression.

**Table 1:** Phenotype of myeloid and dendritic cell subsets under study

L‐DC	Resident monocytes	Inflammatory monocytes	Eosinophils	CD8^+^ cDC	CD8^−^ cDC
CD11c^lo^CD11b^hi^	CD11c^lo^CD11b^hi^	CD11c^−^CD11b^hi^	CD11c^−^CD11b^hi^	CD11c^hi^CD11b^−^	CD11c^hi^CD11b^+^
Ly6C^−^Ly6G^−^	Ly6C^+^Ly6G^−^	Ly6C^hi^Ly6G^−^	Ly6C^+^Ly6G^−^	Ly6C^−^Ly6G^−^	Ly6C^−^Ly6G^−^
CD43^+^SiglecF^−^	CD43^+^SiglecF^−^	CD43^+^SiglecF^−^	CD43^+^SiglecF^+^	CD8^+^	CD8^−^

**Figure 1:**
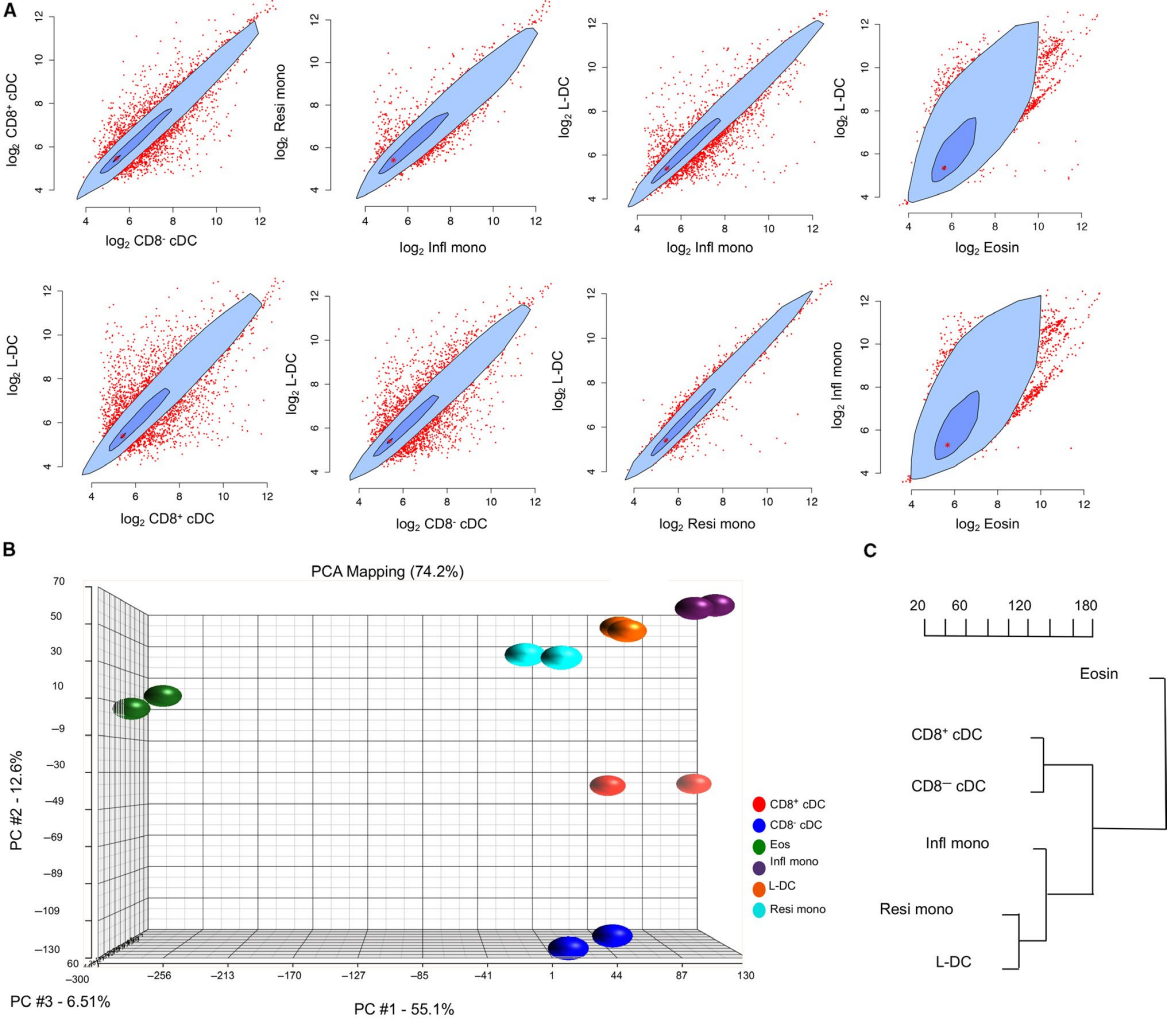
Variability in gene expression amongst dendritic and myeloid subsets. Transcriptome analysis was performed on subsets of cells sorted from murine spleen. RNA was extracted and labelled for hybridization to Murine Gene ST1.0 genechips (Affymetrix). Following scanning to collect signal values from samples prepared in duplicate experiments, data were analysed using Partek and ANOVA by pairwise comparison. A, Mean signal values were calculated and plotted for a total of 35 556 genes in pairwise subset comparisons. The darker blue inner polygon contains 50% of data points, while the pale blue outer polygon contains all other data points which are not outliers (shown in red outside the polygon). The bivariate median is shown by the red asterisk at the centre of the polygon. B, Principle component analysis was used to determine variability in gene expression for each subset. Three principle components are shown for each subset prepared for analysis in duplicate experiments. C, Hierarchical clustering was used to analyse the relationship between subsets on the basis of average gene expression. The dendrogram displays distance between subsets based on clustering of 8508 genes selected for analysis on the basis of mean signal value ≥100 for any one subset

The pairwise relationship between subsets was demonstrated with bivariate plots displaying gene expression in terms of signal value for a total of 35 556 genes (Figure 1A). “L‐DC” and resident monocytes showed the least variance with very few differentially expressed genes evident as outliers (Figure 1A). A comparison of “L‐DC” and inflammatory monocytes showed more differentially expressed genes. The “L‐DC” and cDC subsets showed more variation with many differentially expressed genes evident as outliers. The resident and inflammatory monocyte subsets showed very few differentially expressed genes, indicating a close relationship. The CD8^+^ cDC and CD8^−^ cDC subsets gave a tight bivariate plot with a high number of differentially expressed genes. Eosinophils showed the greatest difference in gene expression in comparison with all other subsets (Figure 1A).

Differences in overall gene expression between the subsets were also evident through principal component analysis (PCA). This showed close grouping of resident monocytes, inflammatory monocytes, “L‐DC” and cDC subsets in the first principal component, but separation of “L‐DC” and monocyte subsets from cDC subsets in the second principal component (Figure 1B). In addition, CD8^+^ cDC were clearly differentiated from CD8^−^ cDC in the second principal component. Lastly, eosinophils were distinct from all other subsets on the basis of the first and second principal components. This analysis indicated similarity between “L‐DC” and monocytes and clearly differentiated “L‐DC” from eosinophils and cDC subsets.

Hierarchical clustering was then used to map the relationship between subsets based on gene expression (Figure 1C). Average signal values from duplicate samples were used for clustering. Genes were included which showed expression in at least one subset (signal value ≥100), giving a sample set of 8508 genes. The analysis indicated a close relationship between “L‐DC” and resident monocytes and then between these two subsets and inflammatory monocytes. CD8^+^ cDC and CD8^−^ cDC were closely related and distinct from the cluster of “L‐DC,” resident monocytes and inflammatory monocytes. As predicted from PCA analysis, eosinophils were quite distinct as a subset and lineage.

### 3.2 Investigation of gene expression specific to subsets

**Figure 2:**
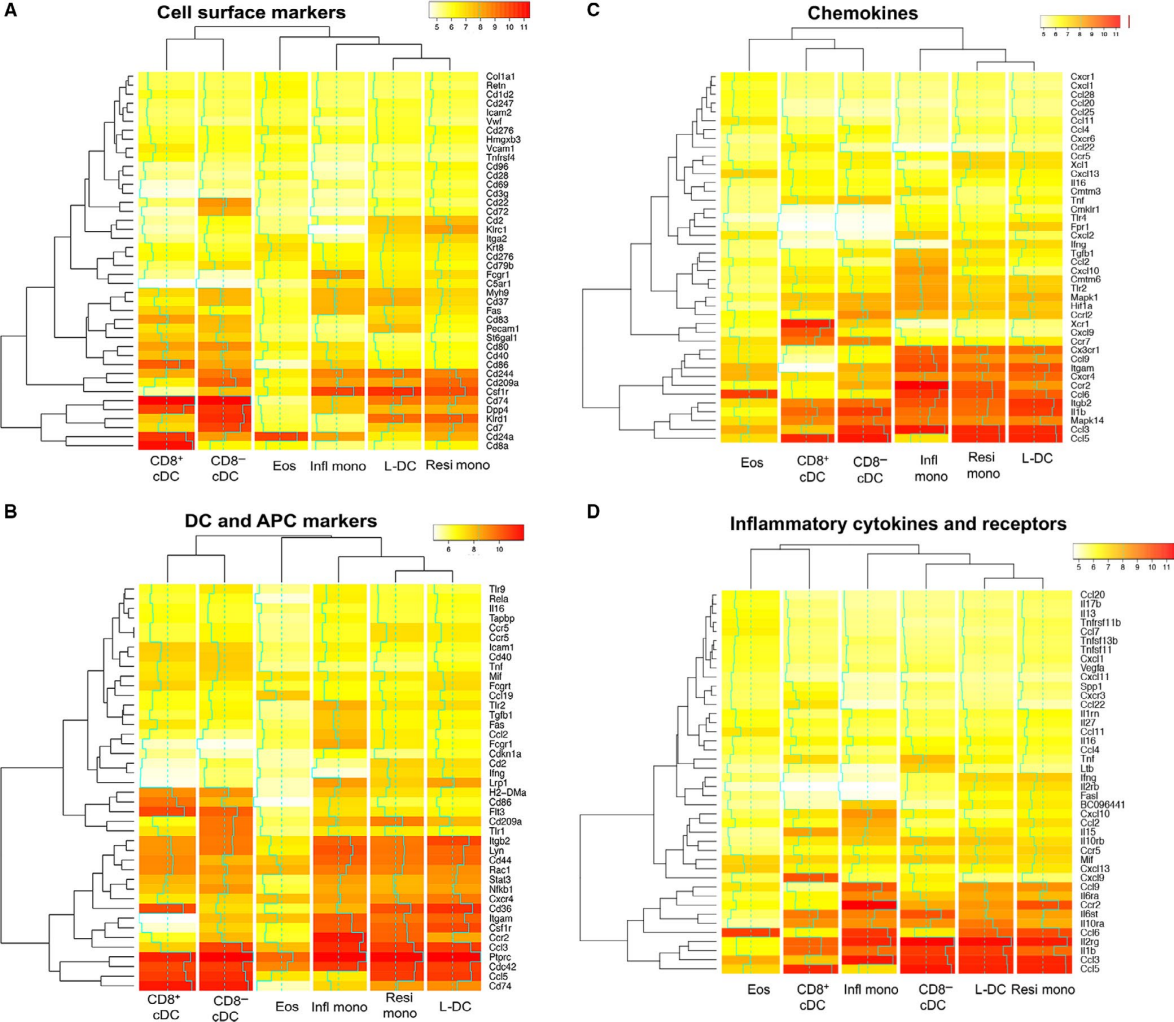
Pathway‐specific gene expression in dendritic and myeloid subsets. Data mining was applied to Affymetrix data sets collected from “L‐DC,” cDC and myeloid subsets prepared in duplicate experiments. For each subset, log_2_ average signal values were plotted as a heat map. The line chart (blue) overlaid on heat maps indicates log_2_ signal intensity changes about the mean (dashed blue line). Genes were clustered by level of expression as shown by row dendrograms. In addition, dendritic and myeloid subsets were clustered on the basis of gene expression as shown by column dendrograms. Data mining involved sets of genes utilized by SABioscience for their PCR arrays. These reflect: (A) Cell surface markers, (B) DC and APC markers, (C) Chemokines and (D) Inflammatory cytokines and receptors

The lineage origin of the different subsets was investigated by data mining and comparing the expression level of known genes to functional categories of “DC and APC,” “Chemokines,” “Cell surface markers” and “Inflammatory cytokines and receptors.” Signal values were collected for sets of 84 genes utilized by SABiosciences (Frederick, MD USA) in their PCR arrays. Data are shown as heatmaps with dendrograms reflecting hierarchical clustering. Gene expression is only shown for genes expressed by at least one subset having a signal value ≥100 (Figure 2A‐D). The CD8^+^ cDC and CD8^−^ cDC subsets showed greatest similarity in gene expression and this was reflected in three of four analyses. These two subsets were reflective of the DC lineage by common high expression of genes encoding cell surface markers including *Cd40*, *Cd74*, *Cd80*, *Cd83*, *Dpp4* and *St6gal1* and genes encoding DC and APC markers including *Flt3*, *H2‐Dma* and *Cdc42* (Figure 2A and 2).^29^, ^30^, ^31^, ^32^ Genes upregulated by CD8^+^ cDC included *Cd8α*, *Cd24*, *Cd86* and *Cd36*, all of which encode known markers of this subset^5^, ^32^, ^33^, ^34^ CD8^−^ cDC showed specific high expression of *Cd7*, *Cd22*, *Cd72*, *Klrd1*, *Cd209a and Tlr1* which are also known markers of CD8^−^ cDC (Figure 2A and 2).^31^, ^32^ In terms of genes encoding chemokines, inflammatory factors and related genes, the two cDC subsets showed common expression of *Ccr7, Ccl5, IL‐1b, Itgb2, IL2rg* and *IL‐10ra* (Figure 2C and 2).^35^, ^36^ CD8^+^ cDC were uniquely marked by expression of *Xcr1* as reported previously,^37^, ^38^, ^39^ as well as expression of *Cxcl9*.^40^ CD8^−^ cDC also specifically expressed *Ccl3* (Figure 2C and 2). These data, and their concordance with descriptions of cDC gene expression in the literature, confirm the efficiency of the cell sorting procedure and gene profiling methodology developed here.

Resident and inflammatory monocytes shared similar gene expression profiles for all functional categories with exceptions amongst chemokines and their receptors and cell surface markers (Figure 2A‐D). Both monocyte subsets expressed *Csf1r* which encodes the receptor for macrophage colony stimulating factor (M‐CSF). In addition, they also expressed *Ccr2* which encodes an essential receptor for monocyte migration.^9^, ^41^ Common expression of *Cxcr3* identifies them as monocyte/macrophage as opposed to dendritic lineage cells.^42^ They also commonly expressed *Itgam, Ccl6, Itgb2, IL1b, Ccl3, IL2rg, Cd244, Kldr1, Lyn, CD44, Cd36, Ptprc* and *Cdc42*. Resident monocytes specifically expressed *Ccl5* and *Cd209a* which encodes DC‐SIGN a binding protein for pathogens commonly expressed by DC.^43^ Inflammatory monocytes specifically expressed *Ccl9*. Eosinophils were distinct from all other myeloid and DC subsets on the basis of their gene expression profile (Figure 2A‐D), expressing high levels of *Ccl6, Cd24a, Ptprc* and *Cdc42*. Weaker expression of *Krt8*, *Ccl19* and *Cxcl13* confirmed their phenotype as reported previously.^44^, ^45^ As eosinophils are shown here to be very distinct from cDC and other myeloid subsets, they have been disregarded from further analysis directed at lineage determination for “L‐DC.”

The “L‐DC” subset showed gene expression more closely linked with resident monocytes than with any other subset across the four functional categories studied. This is shown both in bivariate analysis, PCA and clustering (Figure 1), and by dendrograms above all heatmaps (Figure 2). In addition, inflammatory monocytes were closely clustered with both resident monocytes and “L‐DC.” Genes commonly expressed at high levels across “L‐DC” and both monocyte subsets included *Itgam*, *Cx_3_cr1, Csf1r*, *Itgb2*, *Ccl6, IL1b, Ccl3, Cdc42, Ptprc, CD244* and *IL2rg*. *Itgam* encodes CD11b, a common marker of myeloid cells which mediates the inflammatory response by regulating adhesion and migration of cells to sites of infection.^46^, ^47^ *Cx_3_cr1* encodes a marker common to cells of the myeloid lineage.^48^, ^49^, ^50^

Recently, Gautiar et al (2012) analysed gene expression in different tissue macrophage subsets. That study defined a core signature of 39 genes defining tissue macrophages.^51^ In that study, splenic red pulp macrophages were sorted as F4/80^hi^B220^−^ with the absence of high expression of MHC‐II and CD11c.^51^ This delineation would now be considered too broad incorporating some DC and mo‐DC which express F4/80 and Cd11c. Expression of those 39 genes was determined for the subsets isolated here by data mining, but none of the subsets expressed all 39 genes, and most expressed very few (Figure S1). This suggests that none of the subsets analysed here reflect red pulp macrophages. Further investigation of “L‐DC” has shown it to be readily distinguishable from red pulp macrophages through phenotype^15^, ^17^ and lack of expression markers like ITGA9 and VCAM‐1 and genes like *SpiC* and *Mertk,* previously associated with red pulp macrophages (data not shown).^51^

### 3.3 Genes upregulated in the novel subset

**Figure 3:**
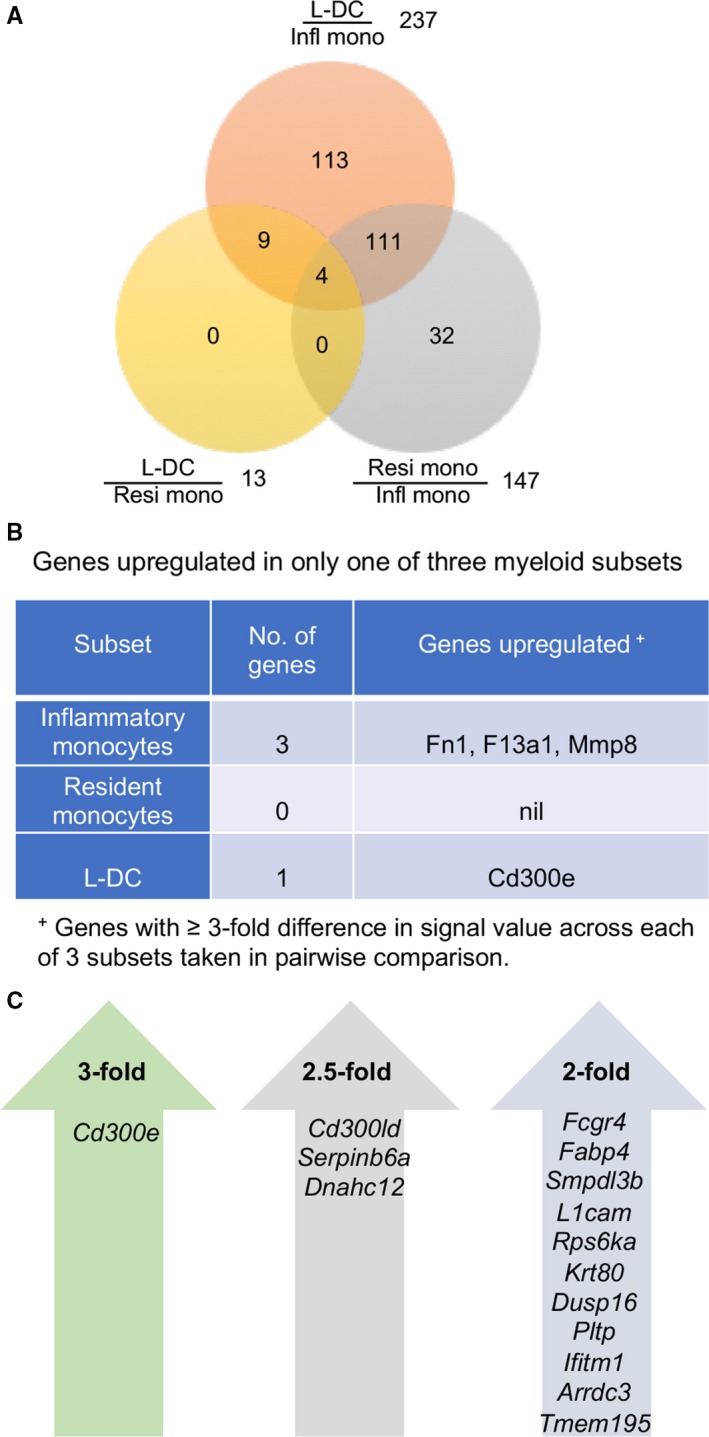
Differential gene expression between “L‐DC” and monocyte subsets. ANOVA was used to make pairwise comparisons of average gene expression (n = 2) between different subsets and to calculate relative fold changes. A, Venn diagram shows numbers of genes upregulated ≥3‐fold in one of two subsets assessed in pairwise comparison. Infl mono: Inflammatory monocytes; Resi mono: Resident monocytes; and “L‐DC.” B, Genes uniquely expressed by each of the three subsets. C, Genes upregulated in ‘L‐DC’ and no other dendritic or myeloid subset in spleen were selected on the basis of mean signal value in “L‐DC” ≥150, with fold change between “L‐DC” and the lowest expressing subset ≥2‐fold, ≥2.5‐fold and ≥3‐fold as shown

Data sets were extracted to identify genes upregulated at least threefold in either “L‐DC” or the two monocyte subsets. “L‐DC” and inflammatory monocytes were found to be the most distinct subsets, while “L‐DC” and resident monocytes were the most closely related (Figure 3A). The data also predict a close relationship between resident monocytes and inflammatory monocytes, consistent with PCA and clustering evidence (Figure 1). Only four genes were found to be uniquely up‐regulated in one of the three subsets. Upregulation of *Fn1*, *F13a1* and *Mmp8* identified inflammatory monocytes, and upregulation of *Cd300e* identified “L‐DC” (Figure 3B). *F13a1* encodes an alternate activation marker for macrophages,^52^, ^53^ *Fn1* encodes fibronectin1 (FN1), involved in cell adhesion, migration and growth and *Mmp8* encodes matrix metalloproteinase‐8 involved in the breakdown of extracellular matrix. FN1, F13A1 and MMP8, known to be specifically upregulated in inflammatory monocytes over resident monocytes.^54^

“L‐DC” were found to be distinct from the two monocyte subsets through at least threefold upregulation of *Cd300e* which is also commonly expressed by all subsets. CD300E is a type I transmembrane protein with a short cytoplasmic tail and a charged transmembrane residue which interacts with DAP12.^55^ It was previously shown to be expressed by macrophages/monocytes, mo‐DC, and at lower levels in in vitro‐derived macrophages and DC.^55^ Upon binding, CD300E induces activation signals calcium mobilization and release of reactive oxygen species by monocytes.^56^, ^57^ In addition, CD300E binding also induces cytokine release by monocytes and promotes survival of monocytes and mo‐DC.^56^, ^57^ DC activated via CD300E have stronger capacity to stimulate T cells.^57^ CD300E upregulation is consistent with the superior antigen presenting capacity of “L‐DC” over the other monocyte subsets.^16^, ^17^ Other genes which were upregulated at 2.5‐fold included *Cd300ld*, *Serpinb6a* and *Dnahc12* (Figure 3C). CD300LD belongs to the same family as CD300E and participates in signal transduction and production of pro‐inflammatory cytokines.^58^, ^59^ *Serpinb6a* encodes a protein essential for protection against cytotoxic granules,^60^ while *Dnahc12* encodes a protein that forms part of dynein.^61^ Genes upregulated ≥2‐fold in “L‐DC” over other subsets were mainly proteases, adhesion proteins and transmembrane proteins. Upregulation of F*cgr4* is of interest because CD300E has been shown to physically interact with FcRγ.^62^

### 3.4 Identification of genes which distinguish the novel subset from resident monocytes

**Figure 4:**
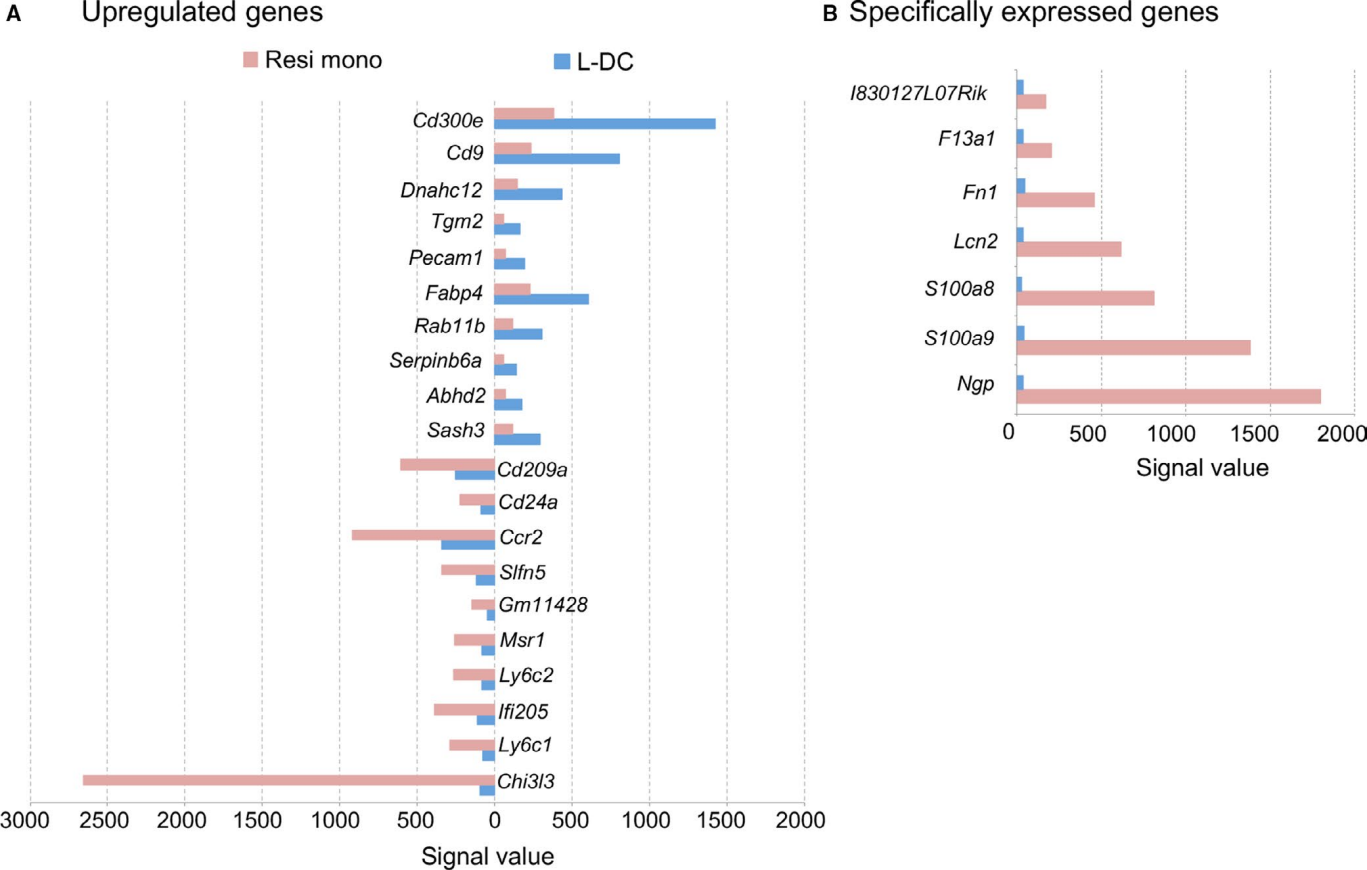
Genes upregulated or specifically expressed between “L‐DC” and resident monocytes. ANOVA was used to make pairwise comparisons of average gene expression (n = 2) between subsets and to calculate relative fold changes. A, Genes upregulated in either “L‐DC” or resident monocytes were selected as those for which the signal value in one subset was ≥50, and the signal value in the second subset was ≥125. Data shown reflect genes with ≥2.5‐fold difference in signal value. B, Genes specifically expressed in either “L‐DC” or resident monocytes were selected on the basis of the mean signal value in one subset ≤50, and mean signal value in the second subset ≥125. No genes were found to be specifically expressed by “L‐DC”

Both PCA and gene expression analyses revealed a close developmental relationship between resident monocytes and “L‐DC” which differ from resident monocytes through low expression of CD43 and absence of Ly6C expression (Table 1). To further investigate this relationship, genes specifically upregulated in either “L‐DC” or resident monocytes were identified (Figure 4). *Cd300e* and *Cd9* were shown to be upregulated in “L‐DC” over resident monocytes, along with *Dnahc12*, *Tgm2*, *Pecam1*, *Fabp4*, *Rab11*, *Serpinb6a*, *Abhd2* and *Sash3* (Figure 4A). Both CD300E and CD9 regulate the ability of DC and monocyte/macrophages to activate T cells.^56^, ^57^, ^63^ In addition, CD9 also modulates cell adhesion and migration^64^ and acts as a potent co‐stimulatory molecule for T cells.^63^ DNAHC12 belongs to the dynein family, comprising proteins that convert energy in ATP into movement,^61^ while FABP4 is involved in T cell priming via regulation of IFN‐γ production by CD8^+^ T cells.^61^, ^65^, ^66^ TGM2 is involved in multiple processes including apoptosis and signal transduction.^67^ RAB11B has been found to participate in both endocytic and exocytic pathways involving Fc receptors to transport intracellular antigens.^68^, ^69^ SERPINB6A is essential for protecting CD8^+^ T cytotoxic lymphocytes against the action of their own cytotoxic granules.^60^ SASH3, also known as SLY1, participates in the regulation of marginal zone B cell development via the Notch signalling pathway.^70^ All of these genes reflect the function of APC and are consistent with the defined functional role of “L‐DC” in CD8^+^ T cell activation and cytotoxic function.^16^, ^17^

Consistent with the reports in the literature, resident monocytes expressed *Ly6c1, Ly6c2* and *Ccr2* (Figure 4A).^9^, ^41^ Expression of Ly6C1/2 reflects the sorting strategy used here, whereby resident monocytes were separated from “L‐DC” (Table 1). In addition, resident monocytes also showed upregulation of several genes known to be expressed by myeloid cells including *Chi3l3*, *Ifi205*, *Msr1*, *Gm11428* and *Cd209a* (Figure 4A).^54^, ^71^, ^72^, ^73^ *Chi3l3* is upregulated ~270‐fold by resident monocytes over “L‐DC.” CHI313 is a marker of alternatively activated M2 macrophages involved in wound healing and tissue repair.^74^ Expression of *Ifi205* regulates the inflammasome adapter protein ASC.^75^, ^76^, ^77^ Macrophage scavenger receptor (MSR1) is involved in the endocytosis of double‐stranded RNA, transportation to endosomes and interaction with TLR3 for triggering IFN responses.^78^, ^79^ As with MSR1 and IFI205, CD209a is expressed by both macrophages and DC.^80^, ^81^ CD209a binds mannose‐type carbohydrates found on viruses, bacteria and fungi, and induces phagocytosis of pathogens by macrophages.^82^ *Gm11428* encodes AMWAP which is expressed by tissue macrophages, microglia and retinal cells and regulates proinflammatory microglia and macrophage activation.^83^ This phenotype distinguishes resident monocytes from the novel APC subset in that it reflects activated monocytes with wound‐healing capacity perhaps related to M2 macrophages.

As very few genes were identified as upregulated by “L‐DC” over resident monocytes, genes specifically expressed by “L‐DC” or resident monocytes were sought. Genes were selected according to the criteria of signal value ≥125 in one subset and ≤50 in the other. This gave a subset of seven genes specific to resident monocytes, but none for “L‐DC” (Figure 4B). Amongst genes specifically expressed by resident monocytes, *Ngp*, *S100a8* and *S100a9* encode monocyte and macrophage markers.^84^, ^85^ NGP regulates monocyte functions of activation and recruitment into sites of infection.^86^ Both S100A8 and S100A9 have been described as activators of endogenous TLR4, so promoting proinflammatory responses.^84^, ^85^ LCN2 is expressed by neutrophils and limits bacterial growth via sequestration of bacterial siderophores containing iron.^87^ Both *Fn1* and *F13a1* are upregulated in inflammatory monocytes over resident monocytes (Figure 4B), but resident monocytes have higher expression of these markers over “L‐DC.” Gene expression analysis, therefore, enabled further distinction between resident monocytes and “L‐DC.” Overall, “L‐DC” show upregulation of many genes with functional roles in antigen processing and presentation to T cells, while resident monocytes show upregulation of genes previously described in relation to monocyte and macrophage function. This evidence supports previous studies showing distinction in terms of marker expression, morphology and capacity to activate T cells.

### 3.5 Identification of markers which distinguish resident and inflammatory monocyte subsets

**Figure 5:**
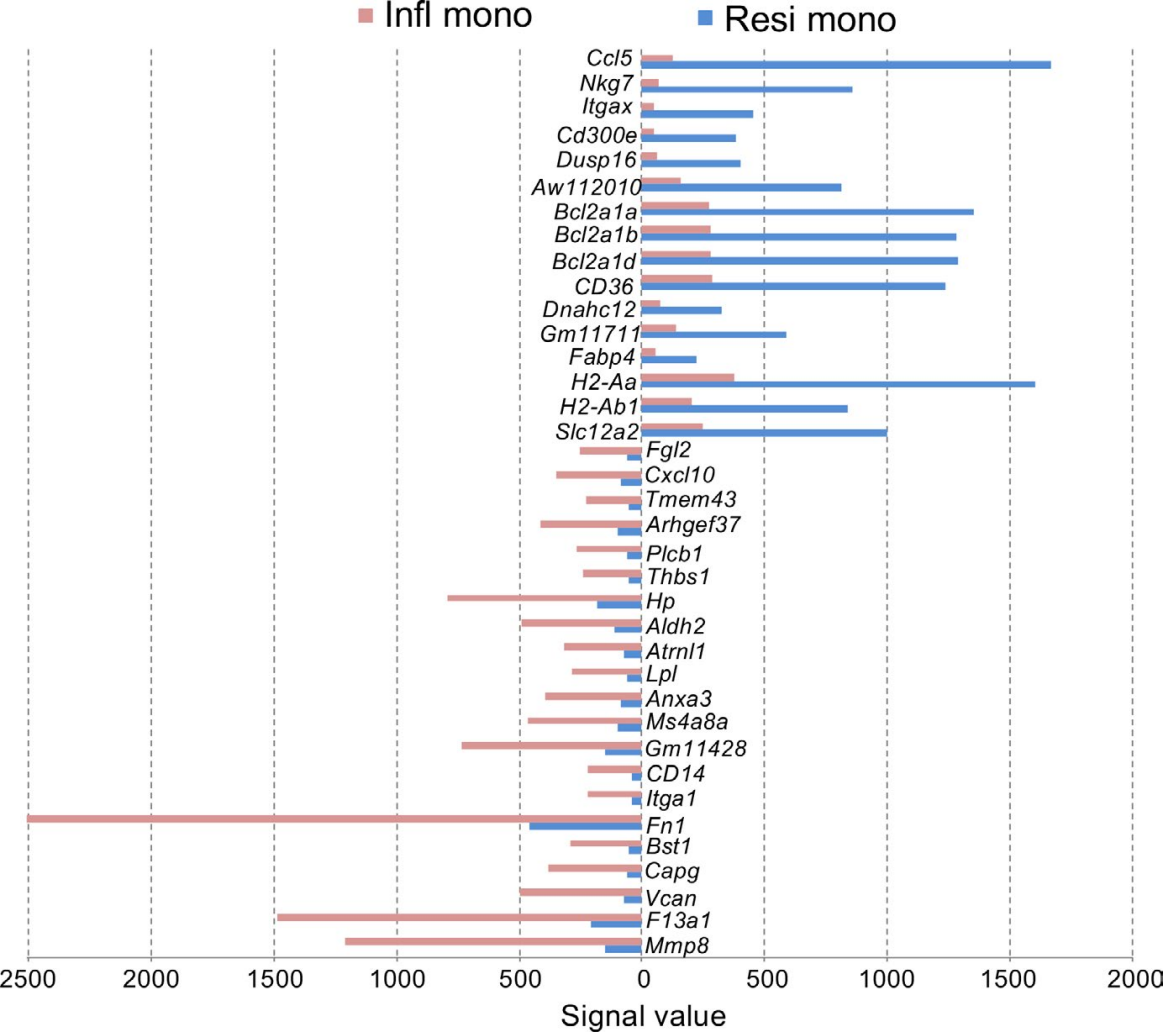
Genes upregulated in either resident monocytes or inflammatory monocytes. ANOVA was used to make pairwise comparisons between average gene expression (n = 2) in inflammatory and resident monocytes. Genes were selected which showed ≥4‐fold change in mean signal value in either resident monocytes (Resi mono) or inflammatory monocytes (Infl mono), where mean signal value in both subsets was ≥50

Both resident (non‐classical) monocytes and inflammatory (classical) monocytes are shown here to be closely related. Previously, blood‐derived inflammatory monocytes were described as precursors of resident monocytes,^88^, ^89^ although that relationship is still unclear. Genes upregulated in one or other subset were therefore identified to further distinguish these two spleen monocyte subsets. In line with earlier gene expression profiles of murine blood monocytes,^54^ resident monocytes from spleen upregulated genes encoding known markers such as *Ccl5*, *Itgax*, *Cd300e*, *Dusp16*, *Cd36*, *H2‐Ab1* and *Fabp4* (Figure 5). Upregulation of *Itgax* (CD11c) by resident monocytes is consistent with the staining and gating strategy used here (Table 1). Upregulation of *H2‐Ab1* and *H2‐Aa* by resident monocytes could indicate potential to express MHCII and act as APC. *Dusp16* is also upregulated and encodes a dual‐specificity phosphatase that can regulate mitogen‐activated protein kinase for signal transduction and gene transcription which selectively regulates cytokine production by myeloid cells.^90^, ^91^ Resident monocytes also show upregulation of *Ccl5* which encodes a chemokine involved in recruitment of leukocytes to sites of inflammation and promotes recruitment and survival of human macrophages.^92^ Inflammatory monocytes were found to upregulate several genes involved in inflammatory monocyte function including *Mmp8*, *F13a1* and *Fn1* described previously, as well as *Vcan*, *Cd14, Capg, Ms4a8a* and *Cxcl10* (Figure 5)*. Cd14* encodes a marker on human inflammatory monocytes which acts as a co‐receptor for TLR4 signalling.^19^, ^93^ *Capg* encodes a protein which participates in control of actin‐based motility in macrophages,^94^, ^95^ *Ms4a8a* encodes a tetraspanin as a marker of activated M2 macrophages,^96^ and *Cxcl10* encodes a chemokine produced mainly by neutrophils and inflammatory monocytes.^97^

### 3.6 Toll‐like signalling during development distinguishes “L‐DC” and resident monocytes

Previously we showed that “L‐DC” can develop in vitro from hematopoietic stem cells (HSC) overlaid above splenic stroma.^27^ During inflammation, the binding of pathogen molecules to Toll‐like receptors (TLR) on HSC can trigger differentiation.^98^, ^99^ The question of whether inflammatory signalling is required for the development of “L‐DC” and other splenic dendritic and monocyte subsets in vivo was therefore addressed. TLR signalling involves the two adaptor proteins MYD88 and TRIF.^100^, ^101^ MYD88 is required for all TLR signalling except TLR3,^100^, ^101^ which uses TRIF for signal transduction and is involved in recognition of double‐stranded RNA associated with viral infection.^102^ TLR4 which binds lipopolysaccharide (LPS) uses both MYD88 and TRIF in association with Toll‐interleukin 1 receptor domain‐containing adapter protein (TIRAP) or Trif‐related adaptor molecule (TRAM), respectively.^100^, ^101^ It has also been shown that dual signalling through MYD88 and TRIF are critical for maximal TLR4‐mediated maturation of DC.^103^

**Figure 6:**
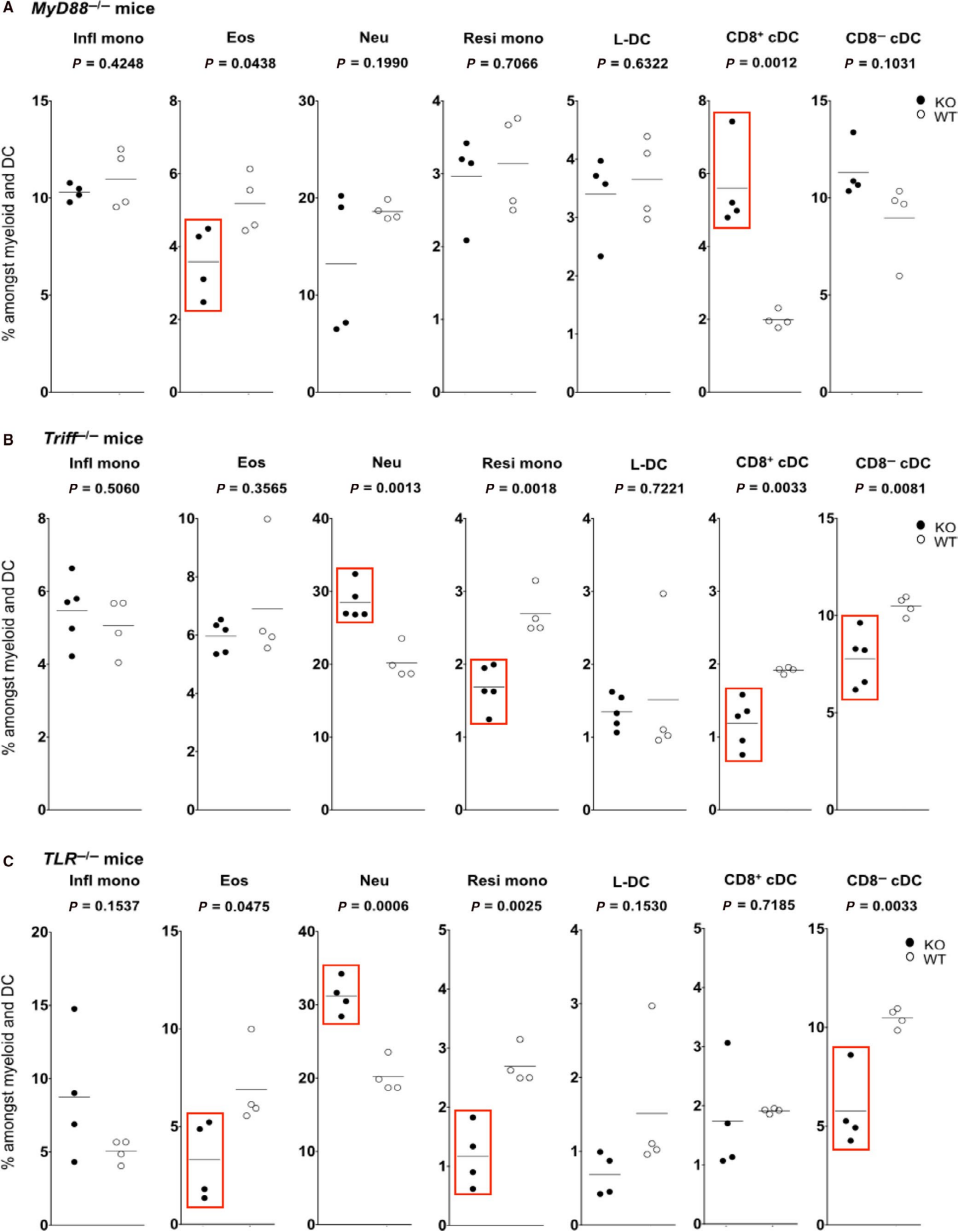
“L‐DC” development occurs independently of Toll‐like receptor signalling. Splenocytes were harvested from C57BL/6J mutant and C57BL/6J (wild type) mice. Cells were stained with antibodies to delineate subsets as described in Table 1. Gates were set based on fluorescence minus one controls, to estimate % cells amongst the total myeloid and dendritic subset (CD11b^+^ and/or CD11c^+^) cells. Individual mice were analysed (n = 4 or 5). A bar is used to show mean values. Wild type mice (Open circles) were compared with mutants (filled circles) (A) *MyD88* ^−/−^ (MyD88 KO) (B) *Trif* ^−/−^ (TRIF KO) and (C) C57BL/6J *MyD88* ^−/−^ *TRIF* ^−/−^ (MyD88/Trif KO). Red boxes indicate significant change in subset representation relative to wild‐type mice using Student’s *t* test (*P* ≤ 0.05)

Analysis of “L‐DC” development in *MyD88* ^−/−^ and *Trif* ^−/−^ mice, therefore, represents a complete test of whether inflammatory signals are essential for the development of “L‐DC” from HSC. The percentage of “L‐DC” and all splenic DC and myeloid subsets amongst the total dendritic and myeloid subset in spleen was, therefore, measured in *MyD88* ^−/−^, *Trif* ^−/−^ and *MyD88* ^−/−^/*Trif* ^−/−^ mice compared with wild‐type control mice. In *MyD88* ^−/−^ mice, a significant 2.5‐fold increase in the percentage of CD8^−^ cDC was seen compared with wild‐type mice (Figure 6A). Eosinophils showed a significant but small decrease. The populations of inflammatory monocytes, resident monocytes, “L‐DC” and neutrophils in *MyD88* ^−/−^ mice were not significantly different from the wild‐type mice. In *Trif* ^−/−^ mice, a significant reduction in percentage of both CD8^+^ cDC and CD8^−^ cDC was observed in *Trif* ^−/−^ mice compared with wild‐type mice and is associated with TLR3 signalling (Figure 6B). Amongst the myeloid subsets, only resident monocytes showed a significant decrease compared with wild‐type mice, while neutrophils demonstrated a significant increase. These data reveal dependency for TLR3 signaling in development of cDC and resident monocytes, but not for “L‐DC” or inflammatory monocytes. The increase in neutrophil number could reflect the higher infection status of these mutant mice, or a compensatory effect. In *MyD88* ^−/−^/*Trif* ^−/−^ double knockout mice which lack all TLR signalling, CD8^−^ cDC showed a significant reduction in number, while there was no change in CD8^+^ cDC (Figure 6C). However, resident monocytes and eosinophils showed a significant reduction, while inflammatory monocytes and “L‐DC” were unaffected (Figure 6C). As with *Trif* ^−/−^ mice, neutrophils showed a significant increase in percentage in *MyD88* ^−^ *^/^* ^−^/*Trif* ^−/−^ over wild‐type mice, which could reflect the infection or inflammatory status of these mice.

From combined studies on the three mutants, it was concluded that TLR signalling is important in the development of CD8^−^ cDC, CD8^+^ cDC, eosinophils and resident monocytes. However, the development of “L‐DC” and inflammatory monocytes occurred independently of TLR signalling, such that the latter two subsets develop in steady‐state spleen. It is important to note that our protocol for delineation of the CD8^−^ cDC subset could also capture inflammatory or mo‐DC whose development would be lost in mutant mice. As “L‐DC” development occurs independently of inflammatory signals, these results serve to distinguish “L‐DC” as a distinct subset from resident monocytes, and to definitively distinguish resident monocytes from inflammatory monocytes.

## 4 DISCUSSION

This study has made a number of contributions towards better understanding dendritic and myeloid subsets present in murine spleen. In particular, a novel subset equivalent to the in vitro generated “L‐DC” cell type has been characterized in terms of gene expression and shown to be distinct from other known DC subsets and monoctyes. Full and complete analysis of splenic subsets initially required that splenic macrophages were first gated out, and that the monocyte subsets were redefined.^15^, ^16^, ^17^ Inflammatory monocytes are now clearly distinguishable as a separate lineage from resident monocytes, and are also distinct from the novel subset of interest. The resident monocyte subset defined in spleen was previously shown to be phenotypically distinct from resident monocytes previously defined in murine blood.^15^ Spleen resident monocytes are now shown to be closely related to a novel APC subset described here as “L‐DC,” such that the two subsets may be derived from a common progenitor or lineage.^104^

The possibility that “L‐DC” reflect a macrophage subset was considered but refuted previously.^17^ It will be necessary in future to better define macrophages amongst dissociated spleen cells on the basis of phenotype because most studies have used immunocytochemical section staining to distinguish these cells. Using flow cytometry, splenic macrophages were here identified as CD11b^lo^CD11c^−^Ly6C^−/+^Ly6G^−^ cells through a series of staining and back‐gating strategies. Further staining of this subset for markers reflecting specific macrophage types then confirmed that “L‐DC” were distinct cells and not macrophages. “L‐DC” did not express MOMA‐1, a marker of marginal metaphyllic macrophages, nor SIGNR1, a marker of marginal zone macrophages.^17^ “L‐DC” do express F4/80 which has been described as expressed by red pulp macrophages and other dendritic and monocyte subsets in spleen. “L‐DC” do not express CD68 as do red pulp macrophages and all other macrophages in spleen. “L‐DC” are also distinct from neutrophils, eosinophils and inflammatory monocytes in terms of phenotype, morphology and gene expression,^15^ and can be delineated from neutrophils through lack of Ly6G and 7/4 expression.^105^, ^106^ “L‐DC” can be also distinguished from eosinophils by lack of Siglec‐F expression,^107^, ^108^ and from inflammatory monocytes through the expression of CD11c and absence of Ly6C expression (Table 1).

Ly6C^hi^ inflammatory monocytes can give rise to mo‐DC‐like TNFα and iNOS‐producing DC (Tip‐DC) in murine tissues during inflammation. Tip‐DC have also been described as classically activated M1 macrophages.^109^, ^110^, ^111^ It is notable that “L‐DC” development occurs independently of inflammatory signals essential for generation of Tip‐DC (Figure 6), and the “L‐DC” phenotype is distinct from that of Tip‐DC through lack of Ly6C and MHCII expression (Table 1). These findings clearly distinguish “L‐DC” from mo‐DC which develop in response to inflammation.

Based on gene expression data obtained here and phenotypic and functional data obtained previously,^15^, ^16^, ^17^, ^112^ “L‐DC” can be distinguished as a unique myeloid subset in spleen. They are more closely related to monocytes than to cDC, although the reason for this could relate to progenitor origin rather than function as an APC. Indeed, their function as APC is distinct from cDC subsets in that they activate only CD8^+^ T cells and not CD4^+^ T cells, and appear to have capacity to cross‐present antigen.^16^, ^17^ The resident (non‐classical) monocyte population in spleen quite distinct from the inflammatory (classical) monocyte subset, despite evidence for a common myeloid phenotype. Both CD8^+^ cDC and CD8^−^ cDC were closely linked in terms of gene profile, and quite distinct from monocytes and the “L‐DC” subset. Lastly, the gene profile of eosinophils was quite distinct from other subsets isolated, suggesting a distinct lineage origin, consistent with evidence that the eosinophil develops from a granulocyte/macrophage progenitor instead of the macrophage/dendritic progenitor.^113^, ^114^, ^115^

Gene profiling studies were conducted with a view to identification of distinguishing markers for “L‐DC” for better classification of this subset. However, markers were not found, and “L‐DC” were shown to be closely related to resident monocytes differing only through upregulation of markers related to T cell activation capacity, namely CD300E, CD300LD, SERPINb6a and CD9. SERPINb6a is widely expressed, and CD300E and CD300LD have expression aligned with DC subsets,^59^ non‐classical monocytes and macrophages.^62^ While no specific genes were found to distinguish “L‐DC” from resident monocytes, a number of specifically expressed genes did distinguish resident monocytes from “L‐DC.” These genes reflect myeloid cells rather than DC including Ly6C, S100A8 and CD209. Although this expression pattern could be consistent with mo‐DC,^116^ no evidence was found for upregulated CD206, or for production of TNF and iNOS, which are delineating markers of mo‐DC.^116^, ^117^

The possibility that “L‐DC” reflect mo‐DC was considered, and refuted on several accounts. Firstly, “L‐DC” do not express markers identified for mo‐DC including SIRPA, S100A8, CD206 and CD209a.^116^ Secondly, “L‐DC” development both in vivo^15^ and in vitro^27^ occurs independently of GM‐CSF, a known inducer of mo‐DC.^117^ “L‐DC” do not express *Stat3a, Stat5a* or *Stat5b* which are important in GM‐CSF‐induced development of mo‐DC (data not shown).^117^ “L‐DC” development in vivo also occurs independently of BatF3, which is important in the development of DC as well as mo‐DC.^118^, ^119^ Development of “L‐DC” in vivo^15^ and in vitro^27^ was shown previously to occur in the absence of inducing cytokines like M‐CSF, GM‐CSF and Flt3L. Cell production also occurs in the absence of c*‐Myb* signalling, suggesting that development does not involve bone marrow‐derived myeloid progenitors but may arise from progenitors endogenous to adult spleen.^112^ “L‐DC” produced in vitro also mirrors an equivalent novel APC subset unique to spleen.

Gene expression profiles obtained here for resident monocytes compared with inflammatory monocytes are consistent with the literature on classical (inflammatory) and non‐classical (resident) monocytes, where both monocyte subsets express *Csf1r* and *Ccr2* and encode receptors essential for monocyte development and migration.^9^, ^41^ Resident monocytes did not show specific gene expression distinguishing them from inflammatory monocytes, although a number of distinct genes were upregulated by each subset. While resident monocytes required TLR signalling for their development, inflammatory (or classical) monocytes are a steady‐state population in spleen, forming in the absence of inflammation. Similarity in gene profile can be attributed to their development from a common lineage origin, or a common progenitor.^48^ Previously, it was reported that blood‐derived Ly6C^hi^ inflammatory (classical) monocytes were a precursor of Ly6C^lo^ resident monocytes (non‐classical and migratory), although data obtained here would not support those findings for similar subsets in spleen.^88^

While the “L‐DC” and resident monocyte populations are closely linked in terms of gene expression, the possibility that they have a precursor‐progeny relationship is refuted on several counts. Firstly, it has always been impossible to drive “L‐DC” to monocytes and *vice versa* through in vitro culture with factors like GM‐CSF, Flt3L or through TLR activation with LPS (Ni, K. & Griffiths, K, unpublished data). In recent studies, however, it was shown that the development of resident monocytes but not “L‐DC” was dependent on Flt3L and GM‐CSF because knockout mice showed loss of resident monocytes but not “L‐DC.”^15^ This suggests that the two cell types follow different pathways for the development, although they may develop from a common progenitor. Data present here in mice which lack TLR‐signalling molecules MyD88 and TRIFF, confirm that finding, showing a loss of resident monocytes but not of “L‐DC.” In contrast, mice mutant for *c‐Myb* show a loss of both resident monocytes and “L‐DC,” consistent with the common progenitor origin.^112^ In vitro studies to define the hematopoietic progenitors which generated “L‐DC” when cocultured above a splenic stromal line which supported hematopoiesis, revealed that “L‐DC” arose only from HSC or multipotential progenitors and not from other myeloid progenitors,^27^ suggesting that “L‐DC” may differentiate directly from HSC in the absence of formation of a myeloid progenitor. Our hypothesis therefore is that “L‐DC” and resident monocytes may have a common progenitor origin in spleen but arise by divergent differentiation.

## 5 CONCLUSION

The close relationship between gene profiles for “L‐DC” and resident or non‐classical monocytes raises questions about a possible common lineage origin. They are shown here to be quite distinct subsets in that resident monocytes require TLR signalling for their development, while “L‐DC” do not. The latter could reflect a steady‐state population of APC in spleen developing in the absence of inflammatory signals. It is yet to be determined whether one of the resident monocyte or “L‐DC” subsets is a precursor of the other, or whether they both reflect functionally distinct progeny of a common progenitor endogenous to spleen. This hypothesis would be consistent with the previous finding that both the “L‐DC” and resident monocyte subsets develop independently of *c‐Myb* expression which distinguishes definitive haematopoiesis and is important for the development of myeloid progenitors in the bone marrow environment.^112^, ^120^

## CONFLICTS OF INTEREST

The authors declare that the research was conducted in the absence of any commercial or financial relationships that could be construed as a potential conflict of interest.

## AUTHOR CONTRIBUTIONS

YH designed, performed and analysed experiments and wrote the paper. TO analysed data and reviewed the paper. HO designed, supervised and analysed experimental work and wrote the paper.

## DATA AVAILABILITY STATEMENT

The authors declare that data will be made available upon request from the authors.
